# Screening for Neuroblastoma Using Urinary Catecholamines: The End of the Story

**DOI:** 10.1093/jncics/pkab042

**Published:** 2021-05-05

**Authors:** William G Woods

**Affiliations:** Aflac Cancer and Blood Disorders Center, Children’s Healthcare of Atlanta/Emory University, Atlanta, GA, USA

Neuroblastoma (NB) is the most common extracranial solid tumor of childhood, with a peak incidence at birth. About 40% of children are diagnosed in the first year of life, and by 6 years of age, more than 90% of children will have presented with the disease. Unlike cancers in adults, where many common malignancies can be preclinically detected, including the 4 most prevalent cancers—lung, colon, prostate, and breast—no pediatric cancer was deemed easily detectable by screening. The 1 exception was NB because it has been known for decades that 90% of patients presenting clinically excrete elevated catecholamine metabolites in their urine. Furthermore, if children present with NB at a young age—specifically, under 18 months of age—the prognosis is outstanding, with about 90% of cases cured. Based on these facts, Sawada and Japanese colleagues pioneered NB screening programs in the 1970s (1), with early results suggesting that overall survival in the children screened improved. Unfortunately, survival can look artificially increased if screening programs diagnose patients whose tumors would have spontaneously regressed, a known phenomenon in NB. One must determine population-based mortality to determine true efficacy of any such intervention.

In the 1980s, several groups around the globe investigated the possibility of instituting NB screening trials, most of which were uncontrolled. In North America, there was a call to begin widespread implementation of newborn screening rather than properly investigating its efficacy (2). Fortunately, other investigators and resources, especially the ability to perform widespread screening, prevailed, and the Quebec NB Screening Study was launched. Geneticists in the Province of Quebec, Canada, had been using 3-week neonatal urine to screen for certain amino-acidopathies that are prevalent in Quebec because of the Founder Effect, with more than 90% compliance (3). The decision was made to screen all children in Quebec at 3 weeks of age and at 6 months of age to mimic the Japanese approach. The province felt that considerable advertising was necessary to convince the public that screening for a childhood cancer was important, especially implementing the new test. Had a randomized trial been done in Quebec, the overlap in who was screened would have been problematic; hence, the use of concurrent geography-based controls (4). About 500 000 neonates could be screened in Quebec over 5 years. In return, several nonscreened control areas with 2 400 000 births, where there were strong population-based registries, were used to assiduously collect data on births, cancers, and deaths over the same 5-year period (1989-1994) (5). During the Quebec NB study, 91% of residents agreed to have their children tested for the cancer at 3 weeks of age, while the compliance at 6 months was 74%. In total, 118 cases of NB were diagnosed vs 54.5 cases expected, more than doubling the incidence (6). Most importantly, the vast majority of cases detected were in children younger than 1 year of age, with no decrease in the incidence after 1 year of age, including patients with advanced-staged disease.

The only other international trial with proper prospective controls was the German NB Screening Project, led by Schilling and colleagues, which made the decision to screen children at 1 year of age, with the thought that the Japanese and American investigators might be screening too early to catch enough cases of NB to make a difference (7). Screening was offered to infants in 6 German states after Unification between 1994 and 1999, while the remaining 10 states served as the control population. Ascertainment of NB incidence and mortality was through the German Childhood Cancer Registry. In total, 61% of the infants were actually screened—a modest number, pointing to the difficulties of introducing a new public health measure in any population. With 2 600 000 children in the screened cohort, their initial results demonstrated like the North Americans that the incidence of NB was doubled, and again no decrease in the incidence of advanced stage disease (7).

In this issue of the Journal, Berthold and colleagues from the German NB Screening Project report the final results of their well-run population-based, controlled project (8). Overall mortality from the disease was determined for the entire study and control area for the 5-year birth cohort ending in 1999, with a minimum of 10 years of follow-up. The authors show no differences in cumulative mortality in the screened and control populations (3.5 vs 3.8 per 100 000 births, *P* = .53). In addition, there were no differences in mortality between the screened cohort and the prestudy or the 5-year poststudy birth cohorts (8). Similarly, in the Quebec study, cumulative mortality was not different between the screened cohort and the control populations (4.8 vs 3.3-5.3 per 100 000 children at 9 years of age) (5). Interestingly, incidence levels decreased to normal after screening in the German study quickly, whereas in the Quebec study, it took 3 years for a drop in NB incidence, representing the “halo effect” (9).

With strikingly similar results in 2 well-run studies, it is more than safe to say that screening for NB using urinary catecholamine metabolites is an ineffective strategy for reducing mortality and should not be implemented anywhere. In recognition of this fact, determined by the German and American studies, the Japanese, who had implemented nationwide NB screening, stopped the mandated practice (10). Why did the preclinical detection of NB not lower mortality? We now know, based on molecular testing, including that on tumor samples from the Quebec study (11), that the vast majority of NB cases detected by screening were good prognosis cases, destined to either spontaneously regress or be cured by surgery and low-dose chemotherapy. In contrast, those NB cases not picked up by screening had a much higher chance of having poor molecular markers, such as elevated MYCN proto-oncogene, and hence poor outcomes. These tumors do not excrete elevated urinary catecholamines; grow so rapidly that screening cannot pick them up; or are resistant to current therapy, even when small. Demonstrating through well-performed studies that such a public health intervention is ineffective, not just to save morbidity and mortality in children but also precious health resources, emphasizes how science is often cost-effective. In the American/Quebec study, it was estimated that between 1989 and 2002—the time period for the entire study—the United States and Canada avoided the unnecessary treatment of 9200 children and false-positive findings in 5000 children and saved $574 million in health-care costs (12).

Berthold et al are to be congratulated for their diligence in giving us long-term mortality data from their NB screening study. This work represents the end of attempts at preclinical detection of a childhood cancer for now. Hopefully, in the future, different approaches can be made using more sophisticated markers to screen children for cancer.

## Funding

Not applicable.

## Notes

**Role of the funder:** Not applicable.

**Disclosures:** The author has no disclosures.

**Author contributions:** Writing, original draft—WGW. Writing, revision and editing—WGW.

## Data Availability

Not applicable.
